# Severe Atelectasis due to Aspirated Valproic Acid Tablet

**DOI:** 10.1155/2024/6650141

**Published:** 2024-03-18

**Authors:** Tomomi Tanigaki, Takunori Ogawa, Sakika Nomura, Koki Ito, Yuhei Kurata, Akira Matsukida, Morio Ishihara, Aihide Yoshino, Akihiko Kawana, Yoshifumi Kimizuka

**Affiliations:** ^1^Division of Infectious Diseases and Respiratory Medicine, Department of Internal Medicine, National Defense Medical College, 3-2 Namiki, Tokorozawa, Saitama 359-8513, Japan; ^2^Division of Psychiatry, National Defense Medical College, 3-2 Namiki, Tokorozawa, Saitama 359-8513, Japan

## Abstract

A 60-year-old man treated with valproic acid (VPA) for epilepsy developed atelectasis and respiratory failure after an accidentally aspirated VPA tablet-induced mucus hypersecretion. Following bronchoscopic removal of the aspirated tablet, his respiratory status improved and massive sputum production did not recur. We hypothesized that the aspirated VPA tablet increased the expression of mucin-related genes, thereby increasing mucus production. Our *in vitro* experiments using a human respiratory epithelial cell line revealed that VPA directly upregulates the airway mucin-related genes. We believe that this is the first case report of aspirated VPA-induced severe atelectasis and respiratory failure, which were successfully treated with the bronchoscopic removal of the VPA tablet.

## 1. Introduction

Mucus secretion is a cardinal airway defense mechanism. The primary physiological function of the secreted mucus is to support the continuous mucociliary clearance of the inhaled particles and pathogens entering the airways [1]. When mucus secretion is abnormal, the excess mucus accumulates in the airway lumen and obstructs the airway [2, 3].

Valproic acid (VPA) is a widely used antiepileptic drug that is administered both orally and intravenously [4]. However, the effects of VPA aspiration remain unknown. We have discussed the case of a patient who was intubated for respiratory failure due to mucus hypersecretion and subsequent severe atelectasis caused by aspirated VPA. An *in vitro* experiment conducted by us also supported this mechanism. We believe this is the first such case report of aspirated VPA-induced severe atelectasis.

## 2. Case Presentation

**Figure 1:**
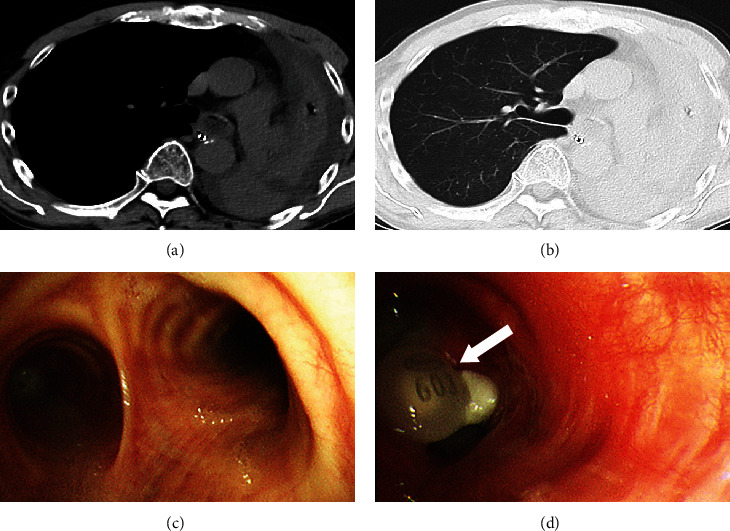
(a, b) Chest computed tomography images demonstrating sputum accumulation in the left main bronchus and a completely atelectatic left lung. Bronchoscopic images showing sputum obstruction of the (c) left main bronchus and the aspirated valproic acid tablet (arrow) in the (d) left inferior lobar bronchus.

A 60-year-old man with no history of respiratory disease was routinely treated with VPA for epilepsy. He was admitted to our hospital for dissociative stupor triggered by the death of his father. He abstained from eating food due to fluctuating levels of consciousness; however, he continued to take a VPA tablet orally because an intravenous infusion of VPA could not be prepared immediately. On the sixth day of hospitalization, he began to expectorate a large amount of white sputum without any signs of fever. Despite frequent suction of the sputum, the sputum quantity continued to increase. Sputum culture did not yield any pathogens; nevertheless, treatment with broad-spectrum antibiotics was initiated on suspicion of bacterial pneumonia. However, his respiratory condition gradually worsened. Three days after the onset of the respiratory symptoms, he was intubated due to severe respiratory failure. His vital signs were as follows: blood pressure, 86/52 mmHg; heart rate, 90 beats per minute; respiratory rate, 24 breaths/minute; and body temperature, 36.1°C. On the day of intubation, chest computed tomography revealed no abnormalities in the right lung but complete atelectasis of the left lung (Figures 1(a) and 1(b)). Laboratory tests revealed a slightly elevated C-reactive protein level (1.1 mg/dL) but normal white blood cell (6,300/*μ*L) and neutrophil (4,599/*μ*L) counts. No pathogens were identified in the sputum culture.

**Figure 2:**
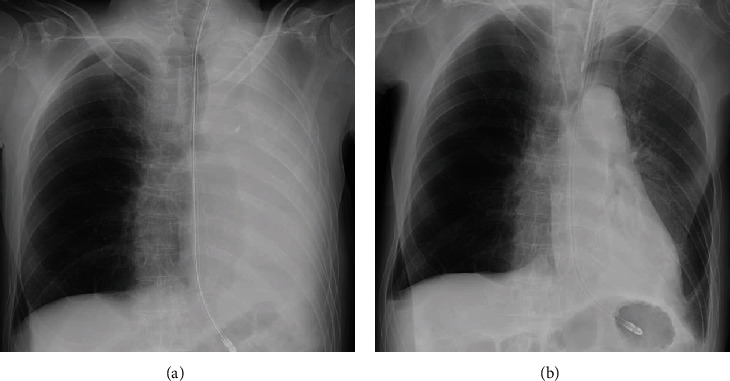
Chest radiographs obtained (a) before and (b) after treatment with sputum aspiration and retrieval of the valproic acid tablet using bronchoscopy.

Bronchoscopy performed after intubation revealed large amounts of white sputum in the left main bronchus and an aspirated VPA tablet in the left inferior lobar bronchus (Figures 1(c) and 1(d)). No obvious bronchial mucosal erosions or erythema was observed. The aspirated VPA tablet was partially dissolved, suggesting leakage of the drug components into the bronchial lumen. The VPA tablet was retrieved using a retrieval basket, and a large amount of sputum was aspirated; thereafter, the atelectasis was resolved completely (Figure 2).

The patient's respiratory status improved immediately after bronchoscopic treatment, and no recurrent massive sputum production or atelectasis was observed. He was extubated after 13 days of intubation, waiting for the subconfusion to improve. Since there were no abnormalities in the swallowing function after extubation, the accidental VPA tablet aspiration was considered to be due to subconfusion.

In the present case, the patient did not have a fever and did not respond to antibiotics; thus, bacterial pneumonia was considered unlikely. We then suspected chemical pneumonia secondary to the aspirated VPA tablet observed on bronchoscopy. Because the infiltrate shadow in the lungs disappeared immediately after the retrieval of the VPA tablet and aspiration of a large amount of sputum, we considered that the lung shadow was not representative of chemical pneumonia but of atelectasis. The aspirated VPA tablet remaining in the bronchial lumen was not large enough to obstruct the main bronchus, suggesting that atelectasis was due to the massive sputum accumulation caused by VPA.

**Figure 3:**
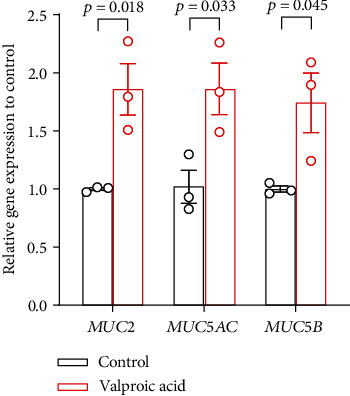
mRNA expression of *MUC2*, *MUC5AC*, and *MUC5B* analyzed using real-time quantitative PCR. NCI-H292 cells were incubated with either the medium alone (control) or with the medium supplemented with valproic acid (5 mM; Cayman Chemical Company #13033) for 24 hours; mRNA was extracted thereafter. Relative gene expression was calculated using the *ΔΔ*Ct method with normalization to *GAPDH*. Data are representative of three independent experiments. Results represent the mean ± standard error of mean. Student's *t*-test was used to compare the groups.

To test this hypothesis, the effect of VPA on the airway epithelium was examined in an *in vitro* experiment using NCI-H292 (a human mucin-producing airway epithelial cell line). NCI-H292 cells were cultured in a medium either alone (control) or supplemented with VPA (Cayman Chemical Company #13033) for 24 hours. The concentration of VPA used in our experiments was based on a previous report [5]. The culture revealed a significant increase in the expression of secretory mucin genes *MUC2*, *MUC5AC*, and *MUC5B* (1.9-, 1.9-, and 1.7-fold upregulation, respectively; Figure 3).

## 3. Discussion

The solid component of airway mucus comprises mucin glycoprotein, which mainly contains mucin 5AC/5B (encoded by *MUC5AC/MUC5B*) [6]. Airway mucus is an innate protective mechanism against toxic chemicals and pathogens and plays a major role in maintaining homeostasis in the lung environment. However, its hypersecretion can cause a progressive decline in the pulmonary function [7].

Epigenetic changes in gene expression lead to an increased or decreased transcription of specific genes without altering the underlying DNA sequence [8, 9]. Several epigenetic controls of histone modifications on mucus production have been reported [10]. 7,4′-Dihydroxyflavone suppresses *MUC5AC* expression in the NCI-H292 cells by upregulating histone deacetylase (HDAC) 2 [11]. The HDAC inhibitors, butyric acid and trichostatin A, upregulate *MUC2/MUC5B* expression in gastrointestinal epithelial cells [12, 13].

VPA is a small branched-chain fatty acid and a potent and widely prescribed drug that acts as an anticonvulsant [14]. It directly inhibits HDACs 1, 2, 5, and 6 [5, 15, 16]. Our *in vitro* study showed that VPA upregulated several mucin-related genes. This finding was consistent with the previously reported effects of other HDAC inhibitors on epithelial cells [13]. If the aspirated VPA increased sputum secretion, the massive sputum production would not have improved unless the VPA tablet in the bronchial lumen was retrieved.

In general, airway foreign bodies are often lodged in the right bronchial tree, because the right main stem bronchus is more vertical [17]. In our case, the tablet was lodged in the left bronchus. The patient was in an unstable state of consciousness and was lying in bed when the tablet was aspirated. Therefore, various factors, including body position, could have caused the tablet to enter the left bronchial tree instead of the right bronchial tree.

This report has several limitations. First, the possibility that contact irritation from the aspirated VPA tablet increased the sputum secretion was not ruled out in this case. In addition, the tablet was not large enough to obstruct the main bronchus alone, but it may have limited the airflow. Severe atelectasis may be caused by these factors in addition to VPA-induced mucus hypersecretion. Second, our *in vitro* experiments revealed that VPA increased the expression of mucin-related genes in airway epithelial cells; however, we did not examine mucin protein secretion. Further experiments are warranted to elucidate the underlying mechanisms.

There have been no reports of VPA-induced severe atelectasis. Our case provides important insights into the appropriate treatment of patients who have aspirated HDAC inhibitors (including VPA).

## Data Availability

The data used to support the findings of this study are included within the article.
